# Naproxen aggravates doxorubicin-induced cardiomyopathy in rats

**DOI:** 10.4103/0253-7613.62411

**Published:** 2010-02

**Authors:** Rahila Ahmad Pathan, Bhulan Kumar Singh, K.K. Pillai, Kiran Dubey

**Affiliations:** Department of Pharmacology, Faculty of Pharmacy, Hamdard University, Hamdard Nagar, New Delhi 110062, India

**Keywords:** Apoptosis, cardiomyopathy, enzyme (kinetics), free radicals, nonsteroidal anti-inflammatory drugs

## Abstract

**Background:**

The repercussion of the heated dispute on cyclooxygenase-2 (COX-2) selective nonsteroidal anti-inflammatory drugs (NSAIDs) led to the national and international withdrawal of several of the recently introduced coxibs. Further debate and research have highlighted risks of the classical NSAIDs too. There is much controversy about the cardiovascular safety of a nonselective NSAID naproxen (NAP) and its possible cardioprotective effect.

**Objectives:**

The study was undertaken to determine the cardiovascular effects of NAP on doxorubicin-induced cardiomyopathy in rats.

**Materials and Methods:**

Male albino rats received a single i.p. injection of normal saline (normal control group) and doxorubicin (DOX) 15 mg/kg (toxic control group). Naproxen was administered alone (50 mg/kg/day, p.o.) and in combination with DOX and DOX + trimetazidine (TMZ) (10 mg/kg/day, p.o.) for 5 days after 24 h of DOX treatment. DOX-induced cardiomyopathy was assessed in terms of increased activities of serum lactate dehydrogenase (LDH), tissue thiobarbituric acid reactive substances (TBARS) and decreased activities of myocardial glutathione, superoxide dismutase and catalase, followed by transmission electron microscopy of the cardiac tissue.

**Results:**

Doxorubicin significantly increased oxidative stress as evidenced by increased levels of LDH and TBARS and decreased antioxidant enzymes levels. Both biochemical and electron microscopic studies revealed that NAP itself was cardiotoxic and aggravated DOX-induced cardiomyopathy and abolished the protective effect of TMZ in rats.

**Conclusions:**

This study indicates that NAP has the potential to worsen the situation in patients with cardiovascular disease. Therefore, it should be used cautiously in patients with compromised cardiac function.

## Introduction

Cyclo-oxygenase-2 selective nonsteroidal anti-inflammatory drugs (NSAIDs), prescribed for the treatment of arthritis and other musculoskeletal complaints are associated with reduced occurrence of gastrointestinal (GI) toxic effects compared with nonselective NSAIDs. However, the VIoxx Gastrointestinal Outcomes Research (VIGOR)[1] and adenomatous colonic polyps (APPROVe)[2] trials and the subsequent withdrawal of rofecoxib owing to an association with increased cardiovascular mortality casts doubt on the cardiovascular safety of other coxibs. It followed logically that the cardiovascular safety of NSAIDs in general should be explored as it had long been known that these drugs cause fluid retention and can increase blood pressure.

Evidence that rofecoxib (Vioxx) increases the risk of myocardial infarction has led to intensive research to assess the risks associated with other coxibs and conventional NSAIDs. At that time many researchers suggested and aggressively pursued the hypothesis that the increased frequency of events was not due to any prothrombic effects of rofecoxib. Non-aspirin, nonsteroidal anti-inflammatory drugs (NANSAIDs) have complex effects that could either prevent or promote coronary heart disease. This research has confirmed that some of these drugs can also increase the risk of cardiovascular events, but the mechanisms and clinical significance are still under intense debate. Results indicating an association between cardiovascular risk and the use of various conventional NSAIDs have recently emerged from some observational studies.[3,4]

Naproxen (NAP) is a NSAID advocated for use in painful and inflammatory rheumatic and certain nonrheumatic conditions. There is no evidence that it is actually cardioprotective. The cardiovascular safety of nonselective NSAIDs has never been systematically studied. Case–control studies have found no cardiovascular effects of NSAID, while a few studies have shown a specific cardioprotective effect for NAP.[5,6] These results contrast with the Alzheimer's disease anti-inflammatory prevention (ADAPT) trial that was recently discontinued, in part because of an excess of cardiovascular events noted with NAP.[7] Therefore, the status of NAP regarding cardiovascular safety till date is still ambiguous.

Doxorubicin, an anthracycline antibiotic, is widely used as effective antineoplastic agent in the treatment of a variety of malignancies, including lymphoma, leukemia, and solid tumors. Unfortunately, the clinical use of this drug is limited by cumulative dose-related cardiotoxicity which may lead to a severe and irreversible form of cardiomyopathy.[8] There are various factors responsible for the development of cardiomyopathy which includes inhibition of nucleic acid and protein synthesis,[9] release of vasoactive amine,[10] abnormalities in mitochondria,[11] formation of free radicals,[12] lipid peroxidation,[12] and depletion of non-protein tissue sulfhydryl groups.[13] However, most studies support the view that an increase in oxidative stress plays a vital role in the pathogenesis of DOX-induced cardiomyopathy.

Trimetazidine is an anti-ischemic drug that restores the ability of the ischemic cells to produce energy and reduces the generation of oxygen-derived free radicals.[14] Various experimental studies have shown that it preserves the intracellular concentrations of ATP and inhibited the extracellular leakage of potassium during cellular ischemia. Additionally, it prevents excessive release of free radicals, which are particularly toxic to phospholipids membranes and are responsible for both the fall in the intracellular ATP concentration and the extracellular leakage of potassium.[15] The prevention by TMZ of DOX-induced myocardial toxicity has been studied on an *in vivo* model in rats.[16] Trimetazidine has been shown to prevent DOX-induced myocardial toxicity by its ability to act as a scavenger of oxygen-derived free radicals, which have been implicated in both early and delayed cardiotoxic manifestations after DOX treatment.[16] In a case study, acute anthracycline-induced cardiotoxic effects resistant to dexrazoxane, was improved after treatment with TMZ.[17]

In this study, DOX treatment was taken as a cardiomyopathy model to investigate the cardiovascular effects of NAP and to compare its effect with TMZ treatment.

## Materials and Methods

### Drugs and chemicals

Doxorubicin HCl (Dabur India Ltd., Sahibabad, U.P., India), naproxen (Ranbaxy Laboratories Ltd., Gurgaon, India,), trimetazidine (Serdia Pharmaceuticals Pvt. Ltd., Mumbai, India), and LDH diagnostic kit (Reckon diagnostics Pvt. Ltd., Vadodara, Gujrat, India) were obtained for the study. All chemicals were of analytical grade and chemicals required for sensitive biochemical assay were purchased from Sigma Chemical Co., USA, Hi Media, and SD Fine Chemicals. Double distilled water was used for all biochemical assays.

### Animals

The study was approved by the Institutional Animals Ethics Committee (IAEC), Hamdard University, New Delhi, India. Fifty-six male albino Wistar rats (250–300 g) were used. They were acclimatized at 25 ± 2 °C under standard laboratory conditions (12 h light and 12 h dark: day and night cycle) and had free access to food and water.

### Experimental protocol

Rats were divided into seven groups, containing eight rats per group. In the normal control group (group 1) (CTR), rats received water for injection. The toxic control group (group 2) received DOX (15 mg/kg single dose) intraperitoneally. The third naproxen per se (NAP PS) group and fourth trimetazidine per se (TMZ PS) group received NAP (50 mg/kg/day, p.o.), and TMZ (10 mg/kg/day, p.o.) for 5 days. The fifth (DOX + NAP) and sixth (DOX + TMZ) groups received, respectively, NAP (50 mg/kg/day, p.o.) and TMZ (10 mg/kg/day, p.o.) for 5 days, 24 h after administration of DOX. In the last group, i.e., DOX + TMZ + NAP group, rats received both the TMZ and NAP for 5 days after 24 h of DOX treatment. After 24 h of last treatment blood samples were withdrawn from the tail vein of rats under light ether anesthesia for biochemical estimation of serum lactate dehydrogenase (LDH).[18,19]

All the animals were then sacrificed by decapitation under light ether anesthesia and hearts were dissected out. Cardiac tissues were washed with ice-cold saline for biochemical estimation of thiobarbituric acid reactive substance (TBARS),[20] glutathione (GSH),[21,22] superoxide dismutase (SOD),[23] catalase (CAT),[24] protein estimation,[25] and for histopathological studies and transmission electron microscopy (TEM).

### Statistical analysis

The results were subjected to analysis of variance followed by Bonferroni's test. *P* values <0.05 were considered statistically significant.

## Results

### General observation and mortality

In the DOX and DOX + NAP groups, the fur of animals became scruffy and developed a light yellow tinge, and there were red exudates around the eyes except in the DOX + TMZ group. All groups of animals except CTR group were suffering from diarrhoea, although more severe diarrhoea was observed in DOX + NAP group. Animals in the DOX-treated group also appeared to be sicker, weaker, and lethargic. The most predominant features in the DOX treatment groups were the development of a grossly enlarged abdomen and ascites.

During the post-treatment period, 37.5% mortality was observed in the DOX + NAP group and 25% mortality was observed in the DOX group. There were no deaths in the CTR group, DOX + TMZ group, NAP PS group, and TMZ PS group. DOX + TMZ + NAP group showed 12.5% mortality.

### Heart weight/body weight ratio

There was a significant (*P* < 0.01) decrease in the heart weight:body weight ratio in the group DOX (2.16 ± 0.94, 1 × 10^−3^) compared to group CTR (2.88 ± 0.47, 1 × 10^−3^). There was no significant fall in the heart weight:body weight ratio in TMZ PS group (2.91 ± 0.81, 1 × 10^−3^), whereas a significant decrease in NAP PS group (2.61 ± 0.35, 1 × 10^−3^) was observed as compared to CTR group. As compared to DOX group, a significant decrease in the heart weight:body weight ratio was found in DOX + NAP group (1.75 ± 0.18, 1 × 10^−3^) but a significant increase was found in DOX + TMZ group (2.72 ± 1.29, 1 × 10^−3^) and DOX + TMZ + NAP group (2.42 ± 0.68, 1 × 10^−3^) [Table 1].

**Table 1:** Heart weight/body weight ratio (×10^−3^) in rats

*Groups*	*HW/BW ratio × 10^−3^*
CTR	2.88 ± 0.47
DOX	2.16 ± 0.94**
NAP PS	2.61 ±0.35*
TMZ PS	2.91 ± 0.81^n.s.^
DOX + NAP	1.75 ± 0.18##
DOX + TMZ	2.72 ± 1.29##
DOX+ TMZ+ NAP	2.42 ± 0.68#

All values are expressed as Mean ± SEM. *n* = 8 rats in each group

* *P* < 0.05

** *P* < 0.01 and nonsignificant (n.s.) when compared to normal control group (i.e., group 1); ANOVA followed by Bonferroni test

# *P* < 0.05 and

## *P* < 0.01, when compared with toxic control group (i.e., group 2); ANOVA followed by Bonferroni test.

### Biochemical parameters

**Serum LDH:** There was a significant increase in serum LDH level in DOX group and DOX + NAP group as compared to normal control (CTR) group (*P* < 0.001) and DOX group (*P* < 0.001), respectively. There was a significant decrease in serum LDH level in the DOX + TMZ group as compared to DOX group (*P* < 0.001). There was a significant increase in LDH levels in NAP PS group (*P* < 0.001) whereas no significant difference was found between TMZ PS group (*P* > 0.05) and normal CTR group. A significant decrease in LDH levels was found in DOX + TMZ + NAP group as compared to DOX group (*P* < 0.05) [Table 2].

**Table 2:** Effect of naproxen (NAP) on serum lactate dehydrogenase (LDH) level in doxorubicin-induced cardiomyopathy in rats

*Groups*	*LDH (IU/L)*
CTR	26.95 ± 1.17
DOX	281.67 ± 0.74*^**^
NAP PS	134.19 ± 1.48**^*^
TMZ PS	27.34 ± 0.74n.s.
DOX + NAP	304.47 ± 1.35##^#^
DOX + TMZ	86.87 ± 1.11##^#^
DOX+ TMZ+ NAP	272.61 ± 3.74#

All values are expressed as Mean ± SEM. *n* = 8 rats in each group

* *P* < 0.05

** *P* < 0.01 and nonsignificant (n.s.) when compared to normal control group (i.e., group 1); ANOVA followed by Bonferroni test

# *P* < 0.05

## *P* < 0.01, when compared with toxic control group (i.e., group 2); ANOVA followed by Bonferroni test.

**Myocardial TBARS:** Tissue lipid peroxides estimated as the level of TBARS were significantly elevated in DOX group as compared to corresponding normal CTR group. There was a significant increase in TBARS levels in DOX + NAP group as compared to DOX group (*P* < 0.001), whereas a significant decrease was found in the DOX + TMZ group as compared to DOX group (*P* < 0.001). There was also a significant increase in TBARS levels in NAP PS as compared to control DOX group (*P* < 0.01) whereas no significant increase in TBARS levels in TMZ PS group was found as compared to control group (group 1) (*P* > 0.05). There was significant decrease in TBARS levels in DOX +TMZ + NAP group as compared to DOX group (*P* < 0.01) [Table 3].

**Table 3:** Effect of naproxen (NAP) on myocardial thiobarbituric acid reactive substance (TBARS), glutathione (GSH), catalase (CAT), and superoxide dismutase (SOD) levels in doxorubicin-induced cardiomyopathy in rats

*Groups*	*TBARS (nmol MDA/mg protein)*	*GSH (μg/ mg protein)*	*CAT (nmol H_2_O_2_/mg protein)*	*SOD (U/mg protein)*
CTR	0.40 ± 0.07	27.70 ± 1.63	18.11 ± 0.97	7.31 ± 0.60
DOX	1.70 ± 0.07***	11.29 ± 0.70***	9.86 ± 0.44***	2.11± 0.21***
NAP PS	0.95 ± 0.17**	18.59 ± 0.62***	11.31 ±0.35***	3.59 ± 0.26***
TMZ PS	0.43 ± 0.09^n.s.^	26.70 ± 0.46^n.s.^	17.96 ± 0.81^n.s.^	8.30 ± 0.17^n.s.^
DOX + NAP	2.60 ± 0.16###	7.61 ± 0.31#	6.75 ± 0.18#	0.62 ± 0.07##
DOX + TMZ	0.75 ± 0.02###	19.81 ± 0.67###	16.02 ± 1.29###	3.83 ± 0.25##
DOX+ TMZ+ NAP	1.07 ± 0.03##	12.88 ± 0.60^a^	13.02 ± 0.68#	2.78 ± 0.45^a^

All values are expressed as mean ± SEM. *n* = 8 in each group.

** *P* < 0.01

*** *P* < 0.001, and nonsignificant (n.s.) when compared to normal control group (i.e., group 1); ANOVA followed by Bonferroni test.

a Nonsignificant.

# *P* < 0.05

## *P* < 0.01, and

### *P* < 0.001 when compared with toxic control group (i.e., group 2); ANOVA followed by Bonferroni test.

**Myocardial GSH:** Tissue GSH level was reduced significantly in DOX group as compared to normal control (CTR) group (*P* < 0.001) [Table 3]. Significant decrease was observed in TBARS levels in DOX + NAP group and as compared to DOX group (*P* < 0.05), whereas a significant increase was found in the DOX + TMZ group as compared to DOX group (*P* < 0.001). There was also a significant decrease in GSH levels in NAP PS as compared to control DOX group (*P* < 0.01), whereas no significant difference in GSH levels in TMZ PS group was found as compared to control group (group 1) (*P* > 0.05). There was no significant difference in GSH levels between DOX + TMZ + NAP group and DOX group (*P* > 0.05) [Table 3].

**Myocardial CAT:** In the DOX group, there was a significant decrease in CAT level compared to normal CTR group (*P* < 0.001). In the DOX + NAP group, there was a significant decrease (*P* < 0.05). However, a significant increase in DOX + TMZ group (*P* < 0.001) was noted as compared to DOX group. There was also a significant decrease in CAT levels in NAP PS group (*P* < 0.001), but no significant difference in cardiac tissue CAT levels was observed in TMZ PS group (*P* > 0.05) as compared to normal CTR group. There was a significant decrease in CAT levels in DOX + TMZ + NAP group as compared to DOX group (*P* < 0.05) [Table 3].

**Myocardial SOD:** Significant reduction of SOD activity was observed in DOX group when compared to normal CTR group (*P* < 0.001). Significant decrease in the SOD level was observed in DOX + NAP group (*P* < 0.01), whereas a significant increase in SOD level was observed in DOX + TMZ group (*P*<0.01). There was also a significant decrease in SOD levels in NAP PS group compared to normal CTR group (*P* < 0.001). However, no significant decrease in TMZ PS group was observed as compared to normal CTR group (*P* > 0.05). There was no significant difference in SOD levels between DOX + TMZ + NAP group and DOX group (*P* > 0.05) [Table 3].

### Histopathological studies

**Figure 1:**
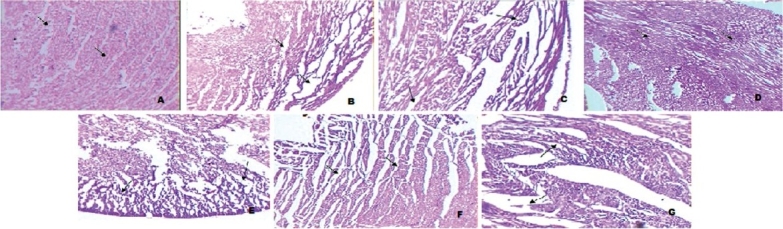
Photomicrograph of rat heart showing normal architecture with regular morphology of myocardial cell membrane and well-preserved cytoplasm (CTR group) (A), doxorubicin (DOX) treated group (DOX) showing subendocardial loss of muscles with inflammatory cells surrounded by edema (B), NAP PS group showed mild edema (C), TMZ PS group showed normal architecture without any pathological symptoms (D), photomicrograph of DOX + NAP group revealed extensive vacuolization, myofibrillar loss and edema (E) whereas DOX + TMZ group and DOX TMZ + NAP group showed mild myofibrillar loss with less extensive vacuolization (F and G) (H and E, ×10).

Histopathological examination of cardiac tissue of normal control group, CTR group, revealed a normal architecture with regular morphology of myocardial cell membrane and well-preserved cytoplasm [Figure 1A]. Marked tissue injury with subendocardial loss of muscles and accumulation of acute inflammatory cells surrounded by mild edema was seen in DOX-treated group [Figure 1B]. Naproxen per se (NAP PS) group showed mild edema [Figure 1C], whereas TMZ PS group showed normal architecture without any pathological symptoms [Figure 1D]. Photomicrograph of DOX + NAP group revealed extensive vacuolization, myofibrillar loss, and edema [Figure 1E], whereas DOX + TMZ group and DOX + TMZ + NAP group showed mild myofibrillar loss with less extensive vacuolization [Figure 1F and G].

**Figure 2:**
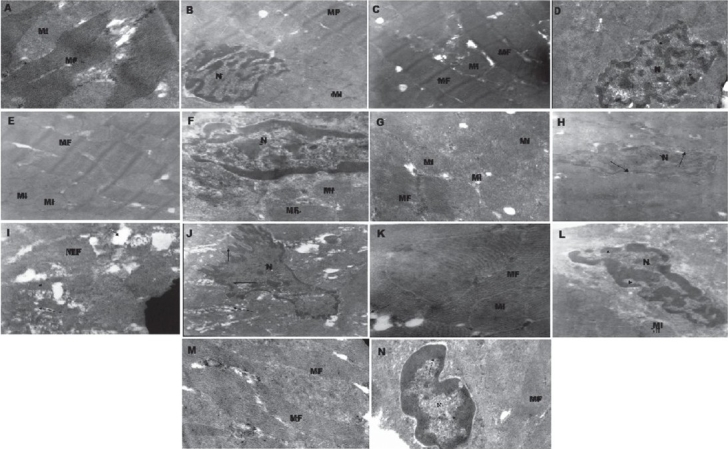
Electron micrograph of rat myocardium of CTR group showed normal mitochondria (MI), myofibrils (MF), and nucleus (N) (A and B; magnification: 0.84 × 10,000 and 0.46 × 10,000, respectively), DOX treated group showing myofibrils with vacuoles and chromatins marginates (arrows) at nuclear membrane (C and D; magnification: 0.84 × 10,000 and 0.46 × 10,000 respectively), NAP PS group showed myofibrils integrity with slightly condensed chromatins marginates (arrows) at nuclear membrane (E and F; magnification: 0.84 × 10,000 and 0.46 × 10,000, respectively), TMZ PS group showed MI, N, and MF integrity (G and H; magnification: 0.84 × 10,000 and 0.46 × 10,000, respectively), DOX + NAP treated group showed cytoplasmic vacuolization, indented nucleus and condensed chromatin marginates at the nuclear membrane (I and J; magnification: 0.84 × 10,000 and 0.46 × 10,000, respectively), DOX + TMZ treated group showed normal mitochondria, myofibril integrity, and less extensive cytoplasmic vacuolization (K and L; magnification: 0.84 × 10,000 and 0.46 × 10,000, respectively), DOX + TMZ + NAP group showed normal myofibril arrangement and condensed chromatin marginates at the nuclear membrane (M and N; magnification: 0.84 × 10,000 and 0.46 × 10,000, respectively).

**Transmission electron microscopical results:** Dramatic morphological changes [Figure 2A and B], including cytoplasmic vacuolization, loss of myofibrils, and nuclear chromatin margination with many condensed pieces of coarse chromatin clumping were observed in DOX group [Figure 2C and D]. However, in all events, the plasma membrane structure was preserved. These morphological changes demonstrate typical myocardial apoptosis.

The NAP PS group [Figure 2E and F] revealed slightly condensed chromatin marginating at the nuclear membrane, whereas mitochondria and myofibrils were found to be normal. TMZ PS [Figure 2G and H] revealed normal nucleus, mitochondria, and myofibrils. Treatment of DOX + NAP treated group [Figure 2I and J] revealed much more condensed chromatin at the margin of the nuclear membrane and extensive cytoplasmic vacuolization as compared to DOX group. DOX + TMZ group [Figure 2K and L] revealed less extensive cytoplasmic vacuolization, with small vacuoles as compared to DOX group. DOX + TMZ + NAP treated group [Figure 2M and N] revealed condensed chromatin at the margin of the nuclear membrane with smaller and more sparsely distributed vacuoles as compared to DOX group.

## Discussion

This study has shown that DOX produced significant cardiomyopathy, as evidenced by increased levels of serum marker enzyme (LDH) and tissue TBARS; decreased levels of myocardial endogenous antioxidants (glutathione, superoxide dismutase, and catalase). Doxorubicin also caused a significant loss of myofibrils and cytoplasmic vacuolization in myocytes.

Doxorubicin-induced cardiomyopathy is related to cumulative dosage. Repeated administration of DOX beyond a certain dose has been shown to cause cardiomyopathic changes in patients[26] and as well as in a variety of animal models.[10,27] Doxorubicin is converted into its semiquinone form in the cardiac myocyte by myocardial CYP450 and flavin monoxygenases. The semiquinone form is a toxic, short-lived metabolite. It interacts with molecular oxygen, initiates a cascade of reactions, and produces reactive oxygen species (ROS).[28] Another reported mechanism of DOX-induced oxidative stress is the formation of a DOX-iron (Fe^2+^) free radical complex.[29] The latter reacts with hydrogen peroxide to produce hydroxyl (OH^·^) radical. ROS reacts with lipids, protein, and other cellular constituents to cause damage to mitochondria and cell membranes of the heart muscle.

Both NAP PS and DOX + NAP groups elevated the levels of serum LDH and cardiac tissue TBARS, whereas it decreased the levels of cardiac tissue superoxide dismutase, catalase, and glutathione as compared to normal and toxic control groups, respectively.

When TMZ was given along with DOX, it decreased the levels of serum LDH, and cardiac tissue TBARS, while increased the levels of cardiac tissue superoxide dismutase, catalase, and glutathione as compared to toxic control group. It showed the protective effect produced by this drug. This may be due to free radical scavenging effect of the drug in the cardiac muscle. Trimetazidine has been shown to protect DOX-induced acute cardiotoxicity by preservation of endogenous antioxidant and reduction of lipid peroxidation.[30]

Trimetazidine when given in combination with NAP and DOX decreased the levels of serum LDH, and cardiac tissue TBARS, whereas increased the levels of cardiac tissue superoxide dismutase, catalase, and glutathione, less significantly as compared to toxic control group thus indicating that NAP interfered with the protective effect of TMZ in DOX model.

Doxorubicin-induced morphological changes in myocardium were observed by electron microscopy. In this study, DOX treatment caused significant histological changes including marked myofibril loss, cytoplasmic vacuolization, chromatin condensation and margination, and membrane blebbing, but maintained the mitochondrial and sarcolemmal integrity.

The NAP PS group revealed condensed chromatin as compared to normal control group. Treatment of NAP along with DOX revealed more condensed chromatin at the margins of the nuclear membrane and extensive cytoplasmic vacuolization as compared to DOX-treated toxic control group indicating aggravation of DOX-induced cardiotoxicity by this group. Treatment of TMZ 10 mg/kg along with DOX exhibited less extensive vacuolization, with smaller and more sparsely distributed vacuoles compared to DOX-treated toxic control group.

Treatment of NAP along with TMZ and DOX revealed condensed chromatin at the margin of the nuclear membrane with smaller and more sparsely distributed vacuoles as compared to DOX-treated toxic control group.

Thus, biochemical and electron microscopic studies together revealed that NAP aggravated DOX-induced cardiomyopathy in rats. In addition, NAP itself was found to be cardiotoxic as revealed by biochemical and confirmed by pathological studies.

Earlier studies believed that NAP was cardioprotective as observed by an unexpected fivefold increase in the risk of acute myocardial infarction (AMI) with rofecoxib when compared with NAP.[1] However, subsequent studies of both rofecoxib and celecoxib also reported an approximate twofold increase in cardiovascular events with both these drugs.[2,31] Ray *et al*. did an observational study to measure the effects of NANSAIDs, including NAP, on risk of serious coronary heart disease and reported no cardiac protection among long-term NANSAIDs users with uninterrupted use.[32] Absence of a protective effect of NAP or other NANSAIDs on risk of coronary heart disease suggests that these drugs should not be used for cardioprotection. Graham *et al*. reported that the use of NAP does not protect against serious coronary heart disease.[33] Huang *et al*. reported that patients with pre-existing medical conditions (e.g., diabetes mellitus, CHF, and dyslipidemia) appeared to have a significantly higher risk for cardiovascular events associated with the use of NSAIDs and celecoxib compared with patients without these conditions.[34] Rahme and Nedjar compared the risk of hospitalization for AMI and GI bleeding among elderly patients using COX-2 inhibitors, NANSAIDs, and acetaminophen, and they reported that among nonusers of aspirin, NAP seemed to carry a greater risk for AMI/GI bleeding whereas among users of aspirin NAP seemed to be least toxic.[35]

Our findings support some of the abovementioned observational studies that NAP itself is cardiotoxic and it is not safe to use this drug in cardiovascular compromised patients.

On the basis of the currently available data, FDA has concluded that the potential for increased risk of serious cardiovascular adverse events is a class effect of NSAIDs. Additional data from long-term, controlled clinical trials are needed to more definitively determine the magnitude of increased risk with NSAIDs, if any.

According to the FDA, all NSAIDs may have similar risks that increase with duration of use and in the presence of existing cardiovascular disease and/or related risk factors. Therefore, clinicians are advised to remain alert for the development of cardiovascular events, even in the absence of previous symptoms and other treatment options should be considered in patients at increased risk for cardiovascular effects.

In conclusion, DOX increased lipid peroxidation and reduced the levels of catalase and GSH in rat heart and caused morphological changes in myocardium, characteristic of apoptosis as shown by TEM and histopathology examination. NAP aggravated DOX-induced cardiomyopathy and itself was found to be cardiotoxic in rats. Trimetazidine showed good protective effect along with DOX but when given along with NAP, its protective effect was abolished. These results suggest that NAP should be used cautiously in patients with cardiovascular disease.
